# ﻿Four new species of Linyphiidae (Arachnida, Araneae) from Hunan Province, China

**DOI:** 10.3897/zookeys.1253.165585

**Published:** 2025-09-26

**Authors:** Wen-long Yan, Feng-qin Wu, Yu-chen Zhou, Xian-jin Peng

**Affiliations:** 1 College of Life Sciences, Hunan Normal University, Changsha, Hunan 410081, China Hunan Normal University Changsha China

**Keywords:** Erigoninae, *
Gongylidioides
*, *
Gongylidium
*, morphology, *
Nippononeta
*, sheet-web spiders, southern China, taxonomy

## Abstract

Four new species of the spider family Linyphiidae Blackwall, 1859 are described from Hunan Province, southern China: *Gongylidioides heimifengensis***sp. nov.** (♂♀), *Gongylidium subbifurcatum***sp. nov.** (♀), *Gongylidium trapezium***sp. nov.** (♀), and *Nippononeta anchorata***sp. nov.** (♀). Detailed descriptions, photos of habitus and copulatory organs, as well as a distribution map, are provided.

## ﻿Introduction

Linyphiidae Blackwall, 1859, commonly known as sheet-web spiders, is the second largest spider family, currently comprising 4949 species in 640 genera (WSC 2025). In recent years, there has been a wealth of research on Linyphiidae of southern China, although relatively less in Hunan Province. Only 40 species of Linyphiidae in Hunan Province have been reported in four papers since 2020 (Irfan et al. 2020; Liu et al. 2023; Gao et al. 2025; Irfan et al. 2025). These studies indicate that Hunan is likely to harbor a substantial diversity of sheet-web spiders which remains undocumented. The present study aims to expand the research on these spiders in Hunan Province. During the examination of spider specimens collected from this province, four new species of linyphiids were detected, which are described and illustrated here.

## ﻿Material and methods

Specimens were stored in 95% ethanol. The left male palps were used for description. The female genitalia were dissected and cleared in lactic acid solution. Specimens were measured and photographed using an Olympus BX53 compound microscope. Focus stack images were generated by Helicon Focus ver. 7.0 and modified by Adobe Photoshop CS5. All measurements are in millimeters. Leg measurements are as follows: total length (femur, patella+tibia, metatarsus, tarsus). The map was created using the online mapping application SimpleMappr (Shorthouse 2010) and modified using Adobe Photoshop CS5. All specimens are deposited at the College of Life Sciences, Hunan Normal University (**HNU**), Changsha, Hunan Province, China. The terminology used in text and figure legends follows Irfan et al. (2025) and Yan et al. (2015).

The following abbreviations are used in the text and figures:

Habitus: **a.s.l.** = above sea level; **AER** = anterior eye row; **ALE** = anterior lateral eye; **AME** = anterior median eye; **AME–ALE** = the distance between AME and ALE; **AME–AME** = the distance between AMEs; **MOA** = median ocular area; **PER** = posterior eye row; **PLE** = posterior lateral eye; **PME** = posterior median eye; **PME–PLE** = distance between PME and PLE; **PME–PME** = distance between PMEs.

Palp: **DTA** = dorsal tibial apophysis; **DSA** = distal suprategular apophysis; **E** = embolus; **L** = lamella; **PC** = paracymbium; **PT** = protegulum; **R** = radix; **RTA** = retrolateral tibial apophysis; **ST** = subtegulum; **SPT** = suprategulum; **T** = tegulum; **TP** = tailpiece.

Epigyne: **CD** = copulatory duct; **CO** = copulatory opening; **DP** = dorsal plate; **FD** = fertilization duct; **S** = spermatheca; **Sc** = scape; **St** = stretcher; **VP** = ventral plate.

## ﻿Taxonomy

**Family Linyphiidae Blackwall, 1859**

### ﻿Genus Gongylidioides

Taxon classificationAnimaliaAraneaeLinyphiidae

Oi, 1960

34705AF5-4637-5097-B84D-C7FAC406F8D5

#### Type species.

*Gongylidioides cucullatus* Oi, 1960.

#### Remarks.

The genus *Gongylidioides* comprises 22 species, including one species known from males and four from females only, distributed in China, India, Japan, Korea, Malaysia (Peninsula), Russia (Far East) and Vietnam. In China, 15 species have been reported (Anhui, Chongqing, Hubei, Hunan, Jilin, Shaanxi, Sichuan, Taiwan, Yunnan and Zhejiang) (WSC 2025).

### ﻿ Gongylidioides heimifengensis sp. nov.

Taxon classificationAnimaliaAraneaeLinyphiidae

FE94266E-AC50-5E12-931C-9ECE5985DE9A

https://zoobank.org/A0D0ED08-0163-4EBE-86B6-BA5A1F8251CF

Figs 1, 2, 3, 4

#### Type material.

***Holotype***: • ♂ (HNU-HMF2303-01), China, Hunan Prov., Changsha City, Wangcheng District, Qiaoyi Town, Changsha Heimifeng National Forest Park, Heimifeng Village; 28.46921°N, 113.00199°E; 98 m a.s.l.; 28 November 2023; X.J. Peng, S.L. Li, Z.Y. Liu, X.Y. Qin, G. Tang leg. ***Paratypes***: • 4♀3♂ same data as for the holotype (HNU-HMF2303-02~03).

#### Etymology.

The specific epithet is derived from the type locality; adjective.

#### Diagnosis.

**Figure 1.**
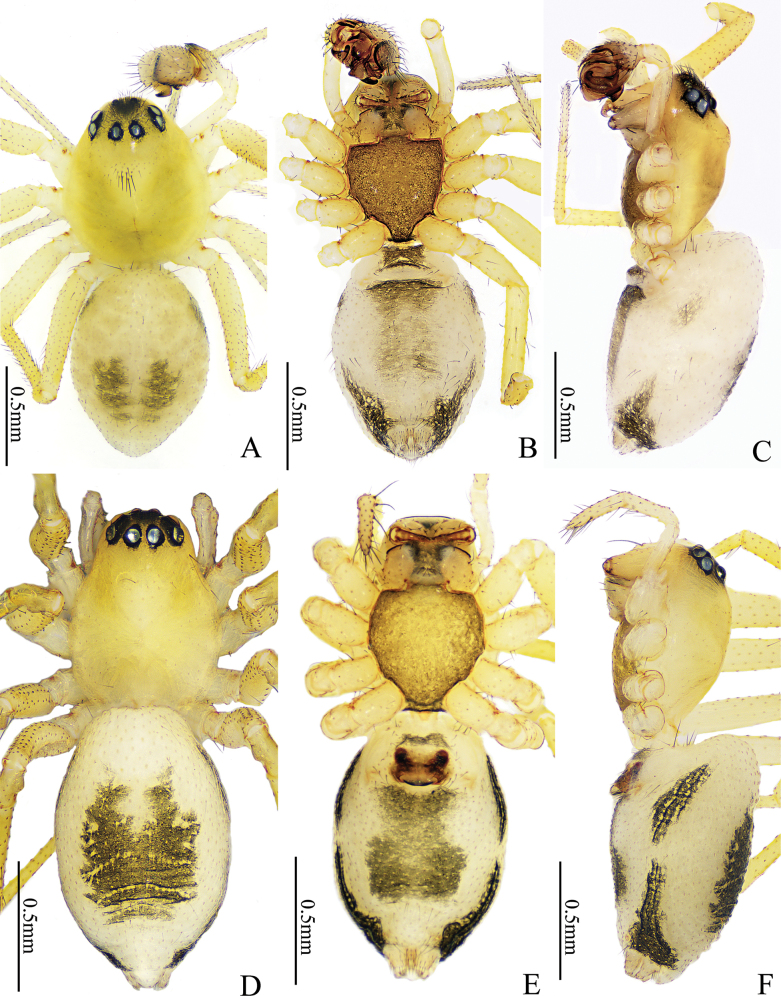
*Gongylidioides heimifengensis* sp. nov., habitus. Male holotype (A–C). A. Habitus, dorsal view; B. Ditto, ventral view; C. Ditto, lateral view. Female paratype (D–F). D. Habitus, dorsal view; E. Ditto, ventral view; F. Ditto, lateral view.

**Figure 2.**
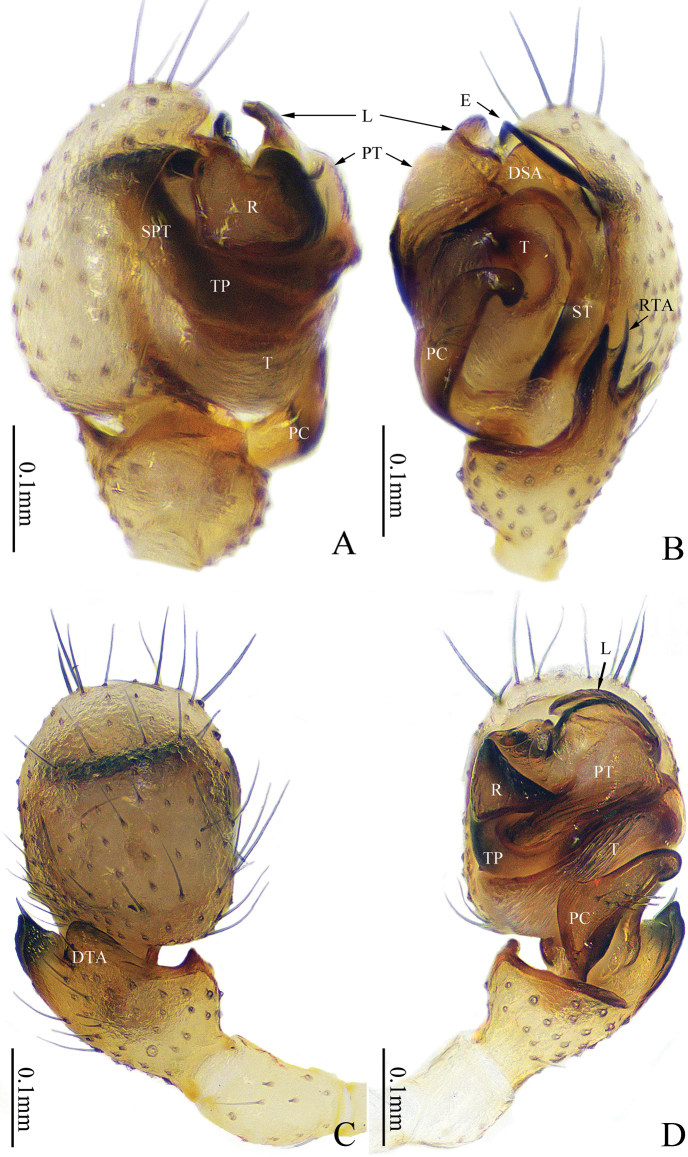
*Gongylidioides heimifengensis* sp. nov., palp of male holotype. A. Palp, prolateral view; B. Ditto, retrolateral view; C. Ditto, dorsal view; D. Ditto, ventral view. Abbreviations: DSA = distal suprategular apophysis; DTA = dorsal tibial apophysis; E = embolus; L = lamella; PC = paracymbium; PT = protegulum; R = radix; RTA = retrolateral tibial apophysis; SPT = suprategulum; ST = subtegulum; T = tegulum; TP = tailpiece.

**Figure 3.**
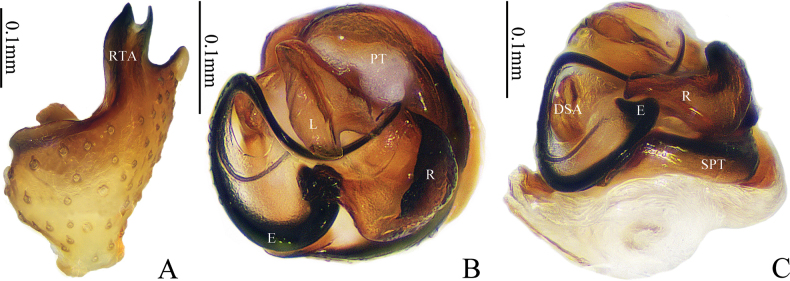
*Gongylidioides heimifengensis* sp. nov., palp of male paratype. A. Tibia, retrolateral view; B. Genitalia bulb, superior view; C. Genitalia bulb, dorsal view. Abbreviations: DSA = distal suprategular apophysis; E = embolus; L = lamella; PT = protegulum; R = radix; RTA = retrolateral tibial apophysis; SPT = suprategulum; ST = subtegulum.

**Figure 4.**
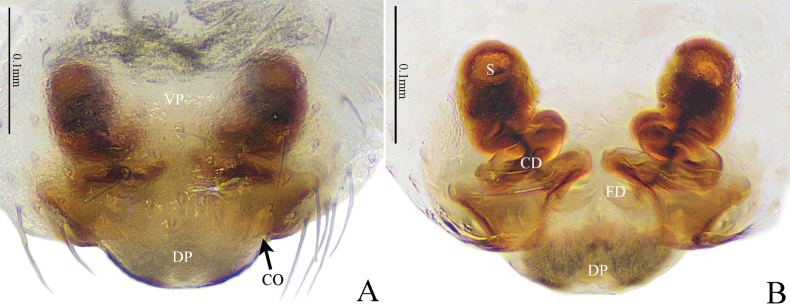
*Gongylidioides heimifengensis* sp. nov., female paratype. A. Epigyne, ventral view; B. Vulva, dorsal view. Abbreviations: CD = copulatory duct; CO = copulatory opening; DP = dorsal plate; FD = fertilization duct; S = spermatheca; VP = ventral plate.

*Gongylidioides heimifengensis* sp. nov. resembles that of *G. cucullatus* Oi, 1960 in having a similar paracymbium (Figs 2, 3; Oi 1960, figs 140, 141), and epigyne with a similar morphology (Fig. 4A, B; Oi 1960, fig. 142), but can be differentiated by (1) carapace slightly elevated in middle (Fig. 1A–C; vs. strongly elevated to a hooded lobe); (2) embolus helical-shaped, forming a coil on distal end of palp (Figs 2, 3B, C; vs. not coiled, extending to ventral of tegulum); (3) tip of retrolateral tibial apophysis bifurcated (Figs 2B, 3A; vs. not bifurcated); (4) dorsal plate semicircular (Fig. 4A; vs. posterior end narrow, tongue-shaped); (5) copulatory ducts helical (Fig. 4B; vs. n-shaped); and (6) spermathecae located at apexes of copulatory ducts anteriorly (Fig. 4B; vs. located dorso-laterally).

#### Description.

**Male** (holotype, Fig. 1A–C). Total length 1.73. Carapace 0.81 long, 0.95 wide; abdomen 1.00 long, 0.68 wide. Carapace oval, slightly yellow; the elevated middle with one small cluster of setae; fovea extremely thin, radial groove absent; AER bent backwards, PER bent forwards; center of ocular area with dense black hairs. Chelicerae pale yellow, with five promarginal and four retromarginal teeth. Endite and labium yellowish brown, sternum blackish brown. Eye sizes and interdistances: AME 0.04, ALE 0.07, PME 0.07, PLE 0.07; AME−AME 0.05, AME−ALE 0.05, PME−PME 0.04, PME−PLE 0.05, ALE−PLE 0.00. MOA 0.17 long, anterior width 0.13, posterior width 0.16. Clypeus height 0.13. Legs light yellow. Leg measurements: I 2.20 (0.83, 0.65, 0.44, 0.28), II 2.37 (0.71, 0.78, 0.52, 0.36), III 2.07 (0.60, 0.62, 0.51, 0.34), IV 2.63 (0.77, 0.86, 0.62, 0.38). Leg formula: 4213. Abdomen oval and white; dorsum and venter with axisymmetric grey maculae; lateral with triangular black maculae surrounding spinnerets. Spinnerets white.

Palp (Figs 2, 3). Patella as long as tibia. RTA finger-like, much longer than wide, with bifurcated tip; DTA short, wider than long, with blunt end. Paracymbium sclerotized, handgun-shaped, with long setae in the middle, hook-shaped apex bending to tibia. Tegulum large, with a triangular transparent protegulum; subtegulum longitudinal, with sclerotized margin; DSA stout. Radix sclerotized and strongly curved, triangular apex bent upward; lamella translucent. Embolus sclerotized, long and helical-shaped; base left-handed spiral and apex right-handed spiral.

**Female** (paratype, Fig. 1D–F). Total length 1.75. Carapace 0.74 long, 0.56 wide; abdomen 1.00 long, 0.65 wide. Eye sizes and interdistances: AME 0.05, ALE 0.06, PME 0.06, PLE 0.07, AME−AME 0.02, AME−ALE 0.03, PME−PME 0.03, PME−PLE 0.04, ALE−PLE 0.01. MOA 0.20 long, anterior width 0.11, posterior width 0.15. Clypeus height 0.08. Leg measurements: leg I 2.45 (0.77, 0.87, 0.50, 0.31); II 2.40 (0.67, 0.82, 0.57, 0.34); III 1.90 (0.57, 0.52, 0.45, 0.36); IV 2.58 (0.77, 0.88, 0.59, 0.34). Leg formula: 4123. Carapace pyriform. Abdomen yellowish white, with two black striped maculae laterally. Other characters as in male.

Epigyne (Fig. 4A, B). Plate about as long as wide. DP semicircular, extending beyond epigynal furrow; CO small. CD helical; helical coils close to each other; spermathecae finger-shaped, located at apexes of CD anteriorly; FD short and translucent.

#### Distribution.

Known only from the type locality (Fig. 9).

#### Variation.

Males (*N* = 4): total length 1.69–1.75; females (*N* = 4): total length 1.51–1.89.

### ﻿Genus Gongylidium

Taxon classificationAnimaliaAraneaeLinyphiidae

Menge, 1868

9FA51D55-E11C-57C0-85BF-1727446BC110

#### Type species.

*Gongylidium nigricans* Menge, 1868; syn. of *Gongylidium rufipes* (Linnaeus, 1758).

#### Remarks.

The genus *Gongylidium* comprises eight species, including one species known from males and three from females only, distributed in China, Italy, Kazakhstan, Russia (Europe to South Siberia), Turkey and Europe. In China, six species have been reported (Guizhou, Hubei, Yunnan) (WSC 2025).

### ﻿ Gongylidium subbifurcatum sp. nov.

Taxon classificationAnimaliaAraneaeLinyphiidae

2C797956-0545-5B58-9E2A-2EDE8D1BD652

https://zoobank.org/E4A584F4-70C8-4F8A-8A64-87316CFABDC9

Figs 5A–C, 6A, B

#### Type material.

***Holotype***: • ♀ (XFS-2202-05), China, Hunan Prov., Shaoyang City, Dongkou County, Jiangkou Town; 27.146777°N, 110.321231°E; 1340 m a.s.l.; 09 July 2022; Y.C. Zhou, S.L. Li, Y Peng, M.T. Zhang, L.F. Li leg. ***Paratype***: • 1♀ (XFS-2202-04), same data as for the holotype.

#### Etymology.

The specific name refers to the similarity of this species to *G. bifurcatum* Irfan, Zhang & Peng, 2022; adjective.

#### Diagnosis.

The female of this new species resembles that of *G. bifurcatum* Irfan, Zhang & Peng, 2022 in having a similar morphology of the epigyne (Fig. 6A, B; Irfan et al. 2022, fig. 115A–C), but can be differentiated by (1) ventral plate well-developed (Fig. 6A; vs. reduced); (2) copulatory ducts C-shaped (Fig. 6B; vs. forming a wave-like loop); and (3) dorsal plate slightly covered by ventral plate (Fig. 6A, B; vs. almost not covered).

**Figure 5.**
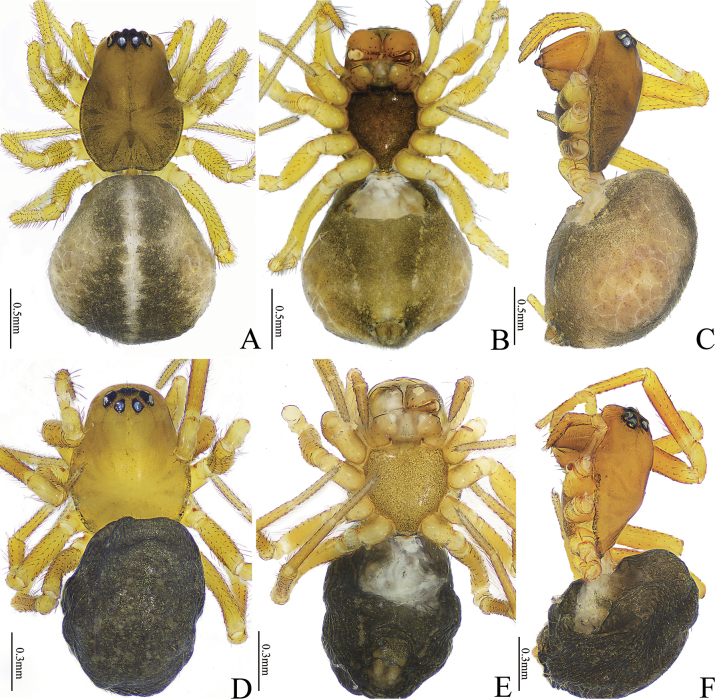
*Gongylidium* species, habitus. *G. subbifurcatum* sp. nov., female holotype (A–C). A. Habitus, dorsal view; B. Ditto, ventral view; C. Ditto, lateral view. *G. trapezium* sp. nov., female holotype (D–F). D. Habitus, dorsal view; E. Ditto, ventral view; F. Ditto, lateral view.

**Figure 6.**
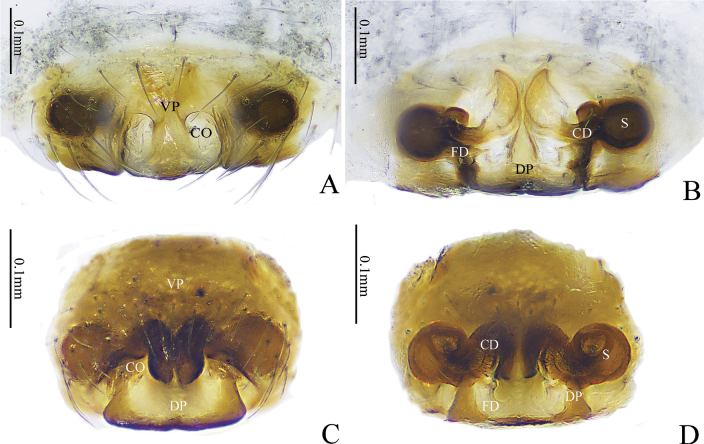
*Gongylidium* species, genitalia. *G. subbifurcatum* sp. nov., female holotype (A, B). A. Epigyne, ventral view; B. Vulva, dorsal view. *G. trapezium* sp. nov., female holotype (C, D). C. Epigyne, ventral view; D. Vulva, dorsal view. Abbreviations: CD = copulatory duct; CO = copulatory opening; DP = dorsal plate; FD = fertilization duct; S = spermatheca; VP = ventral plate.

#### Description.

**Female** (holotype, Fig. 5A–C). Total length 2.70. Carapace 1.16 long, 0.86 wide; abdomen 1.44 long, 1.48 wide. Carapace brown; cervical and radial grooves distinct, fovea indistinct; AER and PER horizontal. Chelicerae light brown, with five promarginal and four retromarginal teeth. Endite and labium light brown; sternum blackish brown. Eye sizes and interdistances: AME 0.06, ALE 0.09, PME 0.08, PLE 0.09; AME−AME 0.01, AME−ALE 0.04, PME−PME 0.05, PME−PLE 0.05, ALE−PLE 0.00. MOA 0.18 long, anterior width 0.12, posterior width 0.20. Clypeus height 0.14. Leg measurements: I 3.30 (0.93, 1.09, 0.73, 0.55), II 2.23 (0.76, 0.66, 0.41, 0.40), III 2.68 (0.78, 0.85, 0.62, 0.43), IV 3.33 (0.96, 1.09, 0.80, 0.48). Leg formula: 4132. Legs yellowish brown. Abdomen pear-shaped; black dorsum longitudinally separated by thin white band in middle, lateral pale brown with scaly patches, venter yellowish brown. Spinneret brown.

Epigyne (Fig. 6A, B). Plate rectangular, about 2× wider than long; septum broad, located at posterior half of plate; CD posteriorly oblique. CD slender, forming one loop before entering spermathecae; spermathecae global; FD short and taper.

**Male**. Unknown.

#### Distribution.

Known only from the type locality (Fig. 9).

#### Variation.

Females (*N* = 2): total length 2.13–2.70.

### ﻿ Gongylidium trapezium sp. nov.

Taxon classificationAnimaliaAraneaeLinyphiidae

03E0DCA5-B7E5-5AA2-89D4-693F442F3F65

https://zoobank.org/42CD5089-5E44-4D68-993F-12D89F32CE2B

Figs 5D–F, 6C, D

#### Type material.

***Holotype***: • ♀ (XFS-2204-03), China, Hunan Prov., Shaoyang City, Dongkou County, Jiangkou Town; 27.146883°N, 110.343392°E; 1294.37 m a.s.l.; 11 July 2022; Y.C. Zhou, S.L. Li, Y Peng, M.T. Zhang, L.F. Li leg.

#### Etymology.

The specific name is derived from the Latin adjective “*trapezium*”, meaning trapeziform, referring to the outline of the dorsal plate in the epigynal plate.

#### Diagnosis.

The female of this new species resembles that of *G. demersum* Irfan, Zhang & Peng, 2025 in having similar copulatory ducts (Fig. 6C, D; Irfan et al. 2025, fig. 96A–C), but can be differentiated by the (1) dorsal plate three times wider than long (Fig. 6C; vs. two times wider than long); and (2) spermathecae located dorso-laterally (Fig. 6D; vs. located anteriorly).

#### Description.

**Female** (holotype, Fig. 5D–F). Total length 1.71. Carapace 0.83 long, 0.62 wide; abdomen 0.88 long, 0.76 wide. Carapace yellowish brown; cervical groove and fovea indistinct; AER horizontal, PER strongly bent forwards. Chelicerae yellowish brown, with six promarginal and five retromarginal teeth. Endite, labium and sternum yellowish brown. Eye sizes and interdistances: AME 0.04, ALE 0.05, PME 0.05, PLE 0.04; AME−AME 0.01, AME−ALE 0.03, PME−PME 0.05, PME−PLE 0.05, ALE−PLE 0.01. MOA 0.14 long, anterior width 0.10, posterior width 0.15. Clypeus height 0.11. Leg measurements: I 1.91 (0.63, 0.63, 0.35, 0.30), II 1.67 (0.52, 0.53, 0.30, 0.32), III 1.44 (0.45, 0.42, 0.30, 0.27), IV 2.01 (0.63, 0.69, 0.38, 0.31). Leg formula: 4123. Legs yellowish brown. Abdomen oval, black; pattern indistinct. Spinneret dark brown.

Epigyne (Fig. 6C, D). Plate oval, slightly wider than long. VP extended outwards and connected to DP, the junction strongly sclerotized; DP trapezoid and sclerotized. CO large, beside the junction and behind VP. CD short and twisty; spermathecae global; FD blurry and hook-shaped.

**Male.** Unknown.

#### Distribution.

Known only from the type locality (Fig. 9).

### ﻿Genus Nippononeta

Taxon classificationAnimaliaAraneaeLinyphiidae

Eskov, 1992

EAA5C6C5-8C16-5A39-AD92-E356BEB302F4

#### Type species.

*Nippononeta kurilensis* Eskov, 1992.

#### Remarks.

The genus *Nippononeta* comprises 25 species, including two species known from males and three from females only, distributed in China, Japan, Korea, Mongolia and Russia (Far East, Kurile Is. and Sakhalin). In China, four species have been reported (Chongqing, Fujian, Guangdong, Guangxi, Guizhou, Hubei, Hunan, Jilin and Shaanxi) (WSC 2025).

### ﻿ Nippononeta anchorata sp. nov.

Taxon classificationAnimaliaAraneaeLinyphiidae

AE37DAC7-5C4D-59B8-A0B0-E59D5569AF4B

https://zoobank.org/A6CC488D-AE37-4D63-A701-F5ADC24D93A8

Figs 7A–C, 8A–D

#### Type material.

***Holotype***: • ♀ (HNU-BLXS2404-19), China, Hunan Prov., Yueyang City, Pingjiang County, Jiayi Town, Beiluoxiao National Forest Park; 28.597247°N, 113.908166°E; 181.33 m a.s.l.; 12 November 2024; S.L. Wang, S.L. Li, G. Tang, W.L. Yan leg. ***Paratypes***: • 3♀ (HNU-HMF2303-4), China, Hunan Prov., Changsha City, Wangcheng District, Qiaoyi Town, Changsha Heimifeng National Forest Park, Heimifeng Village; 28.46921°N, 113.00199°E; 98 m a.s.l.; 28 November 2023; X.J. Peng, S.L. Li, Z.Y. Liu, X.Y. Qin, G. Tang leg. • 1♀ (HNU-XFS-2203), China, Hunan Prov., Shaoyang City, Dongkou County, Jiangkou Town; 27.144129°N, 110.341206°E; 1250.66 m a.s.l.; 10 July 2022; Y.C. Zhou, S.L. Li, Y Peng, M.T. Zhang, L.F. Li leg.

#### Etymology.

The specific epithet is derived from the Latin noun “*anchora*” meaning “anchor” and referring to the junction of the dorsal scape and stretcher, which broadens to an anchor shape.

#### Diagnosis.

The female of this new species resembles that of *N. coreana* (Paik, 1991) in having a similar scape and stretcher (Fig. 8A–D; Irfan et al. 2025, fig. 160A–D), but can be differentiated by the (1) copulatory grooves parallel to each other in dorsal view (Fig. 8A, C; vs. having a somewhat heart-shaped outline); and (2) spermathecae S-shaped (Fig. 8A, C; vs. C-shaped).

#### Description.

**Figure 7.**
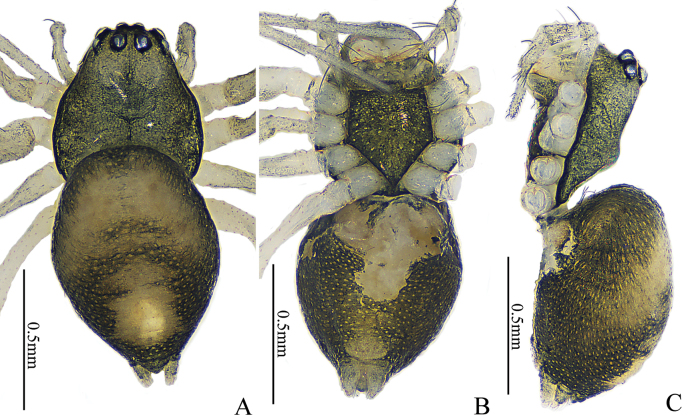
*Nippononeta anchorata* sp. nov., habitus. Female holotype (A–C). A. Habitus, dorsal view; B. Ditto, ventral view; C. Ditto, lateral view.

**Female** (holotype, Fig. 7A–C). Total length 1.15. Carapace 1.62 long, 0.53 wide; abdomen 0.78 long, 0.59 wide. Carapace blackish brown; cervical and radial grooves distinct, fovea thin; AER and PER horizontal. Chelicerae pale, with five promarginal and five weak retromarginal teeth. Endite and labium pale; sternum blackish brown. Eye sizes and interdistances: AME 0.04, ALE 0.05, PME 0.06, PLE 0.05; AME−AME 0.02, AME−ALE 0.03, PME−PME 0.04, PME−PLE 0.02, ALE−PLE 0.01. MOA 0.13 long, anterior width 0.11, posterior width 0.16. Clypeus height 0.08. Leg measurements: I 2.06 (0.59, 0.69, 0.41, 0.37), II 1.96 (0.55, 0.63, 0.44, 0.34), III 1.57 (0.44, 0.50, 0.36, 0.27), IV 2.09 (0.62, 0.68, 0.47, 0.32). Leg formula: 4123. Legs pale white. Abdomen oval; black dorsum with one large pale macula. Spinneret pale grey.

**Figure 8.**
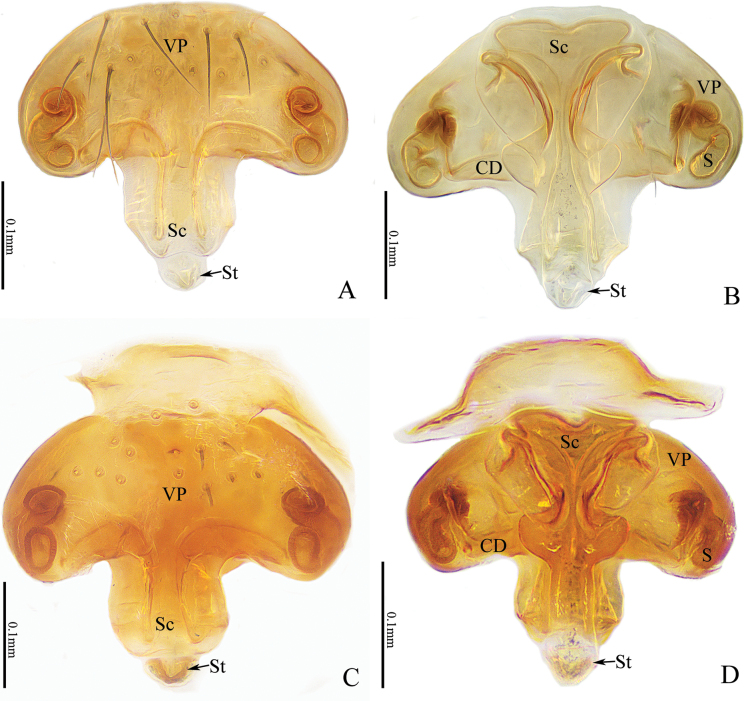
*Nippononeta anchorata* sp. nov., genitalia. Female holotype (A, B). A. Epigyne, ventral view; B. Vulva, dorsal view. Female paratype (C, D). C. Epigyne, ventral view; D. Vulva, dorsal view. Abbreviations: CD = copulatory duct; FD = fertilization duct; S = spermatheca; Sc = scape; St = stretcher; VP = ventral plate.

Epigyne (Fig. 8A–D). Plate inverted anchor-shaped, as wide as long; VP kidney-shaped, about 2× wider than long. Scape sigmoid folded; ventral scape slender, broadened at turning point; dorsal scape inverted triangular. The junction of dorsal scape and stretcher well broadened to anchor shape; CD right-angled; sigmoid spermathecae located at the lateral margin of ventral plate.

**Male.** Unknown.

#### Distribution.

**Figure 9.**
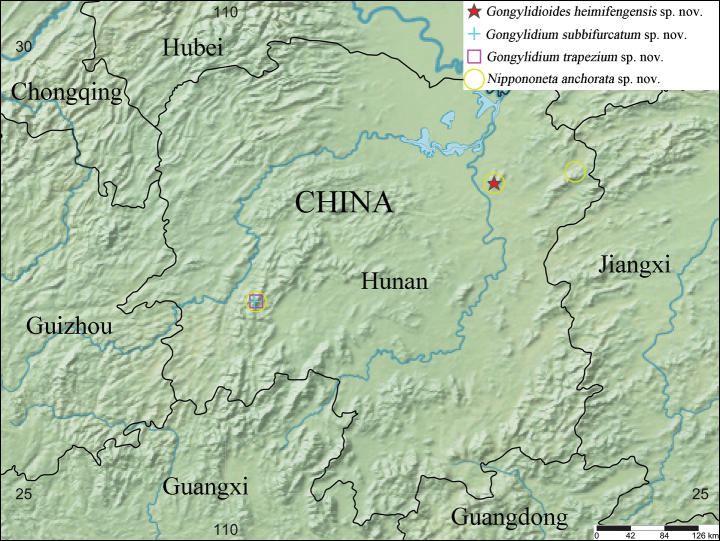
Distribution of the four new species described in this study.

China (Hunan) (Fig. 9).

#### Variation.

Females (*N* = 5): total length 1.15–1.41.

## Supplementary Material

XML Treatment for Gongylidioides

XML Treatment for Gongylidioides heimifengensis

XML Treatment for Gongylidium

XML Treatment for Gongylidium subbifurcatum

XML Treatment for Gongylidium trapezium

XML Treatment for Nippononeta

XML Treatment for Nippononeta anchorata

## ﻿Additional information

### Use of AI

No use of AI was reported.

### Author ORCIDs

Wen-long Yan https://orcid.org/0009-0001-9663-9436

Feng-qin Wu https://orcid.org/0009-0000-1749-0606

Yu-chen Zhou https://orcid.org/0009-0007-7540-1780

Xian-jin Peng https://orcid.org/0000-0002-2614-3910

### Data availability

All of the data that support the findings of this study are available in the main text.
